# Regulation of T cell function by protein S-acylation

**DOI:** 10.3389/fphys.2022.1040968

**Published:** 2022-11-16

**Authors:** Savannah J. West, Darren Boehning, Askar M. Akimzhanov

**Affiliations:** ^1^ Department of Biochemistry and Molecular Biology, McGovern Medical School, University of Texas Health Science Center at Houston, Houston, TX, United States; ^2^ MD Anderson Cancer Center and University of Texas Health Science at Houston Graduate School, Houston, TX, United States; ^3^ Department of Biomedical Sciences, Cooper Medical School of Rowan University, Camden, NJ, United States

**Keywords:** S-acylation, palmitoylation, protein acyltransferases, palmitoyl acyltransferases, T cell receptor, T cell signaling, DHHC enzymes

## Abstract

S-acylation, the reversible lipidation of free cysteine residues with long-chain fatty acids, is a highly dynamic post-translational protein modification that has recently emerged as an important regulator of the T cell function. The reversible nature of S-acylation sets this modification apart from other forms of protein lipidation and allows it to play a unique role in intracellular signal transduction. In recent years, a significant number of T cell proteins, including receptors, enzymes, ion channels, and adaptor proteins, were identified as S-acylated. It has been shown that S-acylation critically contributes to their function by regulating protein localization, stability and protein-protein interactions. Furthermore, it has been demonstrated that zDHHC protein acyltransferases, the family of enzymes mediating this modification, also play a prominent role in T cell activation and differentiation. In this review, we aim to highlight the diversity of proteins undergoing S-acylation in T cells, elucidate the mechanisms by which reversible lipidation can impact protein function, and introduce protein acyltransferases as a novel class of regulatory T cell proteins.

## 1 Introduction

Protein S-acylation is the reversible post-translational lipidation of cysteine residues *via* a labile thioester bond. It was discovered in 1979 when Schmidt and Schlesinger used thin-layer and gas-liquid chromatography to identify the post-translational addition of palmitic acid to the glycoprotein of the vesicular stomatitis virus (Schmidt and Schlesinger, 1979). Since many subsequent studies used tritiated palmitic acid or other palmitic acid analogs to monitor protein S-acylation, it is also often referred to as S-palmitoylation or, simply, palmitoylation. However, palmitate is not the only long-chain fatty acid that can reversibly modify cysteine residues. It has been found that a variety of saturated (myristate and stearate), monounsaturated (oleate), and polyunsaturated (arachidonate and eicosapentanoate) fatty acids can also modify proteins *via* the same thioester bond (Fujimoto et al., 1993; Muszbek and Laposata, 1993; Hallak et al., 1994; DeMar and Anderson, 1997; Montigny et al., 2014). Due to the recent advances in proteomics approaches to detect protein S-acylation, the number of S-acylated proteins is rapidly growing and it has been estimated that at least 20% of the total human proteome (and more than 50% of transmembrane T cell proteins) is targeted by this modification (Morrison et al., 2015; Blanc et al., 2019).

S-acylation is a distinct form of protein lipidation since unlike other types, such as N-myristoylation or prenylation, it is reversible and the modified proteins can undergo multiple cycles of S-acylation and de-acylation (Chamberlain and Shipston, 2015). The reversible nature of the thioester bond suggests that this modification can serve as a unique signaling mechanism coordinating propagation of a signaling cascade through rapid changes in protein hydrophobicity. Indeed, we and others demonstrated the essential role of S-acylation in regulation of the critical proximal components of the T cell receptor (TCR) signaling machinery, such as Lck, Fyn, ZAP-70, LAT, and Orai1/STIM1 (further discussed below).

Although S-acylation was discovered in 1979, there have been several limitations that have hindered the progression of the field until somewhat recently, including identification of the enzymes that catalyze reactions of S-acylation and de-acylation, and the lack of efficient methods to detect protein S-acylation. It was not until 20 years after the discovery of S-acylation that the proteins facilitating this modification, protein acyltransferases, were identified in yeast. Work in *Saccharomyces cerevisiae* was critical for the field when it was uncovered that Erf2/Erf4 and Akr1 were responsible for the S-acylation of Ras2 and Yck2, respectively (Bartels et al., 1999; Lobo et al., 2002; Roth et al., 2002). These enzymes were found to have cysteine-rich domains along with a catalytic consensus sequence of Asp-His-His-Cys, giving them the name zDHHC enzymes. The discovery of zDHHC enzymes in yeast then led to studies finding them conserved in all eukaryotic organisms. Yeast have up to seven zDHHC family protein acyltransferases, while *C. elegans* and *Drosophila* are predicted to have twenty, mice have twenty-four, and humans express twenty-three zDHHC enzymes (Roth et al., 2002; Ohno et al., 2006; Bannan et al., 2008).

Another limitation the field faced was the lack of identified de-acylating enzymes. In 1993, almost twenty years after the discovery of S-acylation, Camp and Hofmann found that palmitoyl protein thioesterase 1 (PPT1) enzymatically removes the acyl group from S-acylated H-Ras (Camp and Hofmann, 1993). However, it was later shown that PPT1 primarily removes fatty acids from proteins being broken down in the lysosomal lumen, and was not localized to the cytosol where zDHHC-mediated S-acylation occurs (Verkruyse and Hofmann, 1996). Even though this enzyme contains a thioesterase signature, including a Ser-His-Asp catalytic sequence and an α/β hydrolase fold, it was not a candidate for protein de-acylation (Camp et al., 1994; Bellizzi et al., 2000), and the enzymes mediating de-acylation remained enigmatic. Then in 1998, it was found that acyl-protein thioesterase 1 (APT1), a member of the metabolic serine hydrolase superfamily, is a cytosolic enzyme and can de-acylate G_α_s *in vivo* (Duncan and Gilman, 1998). APT1 was the first verified enzyme that reverses protein S-acylation. Following the finding of APT1, bioinformatics studies were done to search for other APTs. They led to the discovery of APT2 which de-acylates proteins such as growth associated protein 43 (GAP-43) and H-Ras (Tomatis et al., 2010; Lin and Conibear, 2015), and for several years, APT1/2 were the only enzymes known to reverse S-acylation. In 2015, Lin and Conibear, used a combination of elegant biochemical approaches to demonstrate that members of the ABHD17 family (also part of the metabolic serine hydrolase superfamily) can de-acylate N-Ras and PSD-95 proteins (Lin et al., 2015). This finding was independently confirmed by another group which showed that ABHD17 thioesterases control PSD-95 S-acylation and its synaptic function (Yokoi et al., 2016). These observations indicate that the family of de-acylating enzymes is likely much larger than originally thought and more work is expected to be done in identifying the enzymes responsible for protein de-acylation.

The final problem that delayed the S-acylation field was the lack of effective assays. Although S-acylation was discovered in 1979, it was not until the early 2000’s that there were safe and efficient ways to assay protein S-acylation. Prior to this, it was mostly assayed using radioactively labeled fatty acids and autoradiography to visualize fatty acid incorporation (Schlesinger et al., 1980). This involved using radiation and exposure times that could last weeks to months. Now, we have much safer and faster assays including metabolic labeling (Hannoush and Sun, 2010), PEG-shift (Burgoyne et al., 2013), acyl-biotin exchange (Drisdel and Green, 2004), and acyl-resin assisted capture (Tewari et al., 2020). Additionally, although there is no consensus sequence to fully predict protein S-acylation, there are databases containing information on S-acylated proteins, such as SwissPalm and CSS-Palm, as well as a bioinformatics study that used machine learning algorithms to identify potential S-acylation sites (Ren et al., 2008; Blanc et al., 2019; Li et al., 2021). However, since these approaches and others, have been recently reviewed in depth (Chen et al., 2021), they will not be thoroughly discussed here.

Despite the initial obstacles which delayed the advancements of the field, it has now become clear that S-acylation is a widespread post-translational modification with a broad range of important regulatory functions. Dysfunctions of zDHHC enzymes have been shown to cause a variety of human disorders, such as Huntington disease, cystic fibrosis, schizophrenia, and several types of cancer [reviewed in (De and Sadhukhan, 2018)]. Recently, it has been found that S-acylation is required for the proper function of the immune system. For example, several key signaling T cell receptor proteins were identified as S-acylated, and this modification has been found to be critical for proper protein localization, function, and propagation of T cell receptor signaling in response to antigenic stimulation (Tewari et al., 2021; Fan et al., 2020; Shayahati et al., 2021). Although a lot of progress has been made in the S-acylation field in the last decade, the full extent of S-acylation in T cells is still not fully understood or appreciated. This review aims to demonstrate a broad variety of S-acylated T cell proteins, elucidate the effects of S-acylation on protein function and TCR signaling, and introduce zDHHC/APT enzymes as novel regulators of the immune system.

## 2 Proximal T cell signaling and the immunological synapse

**FIGURE 1:**
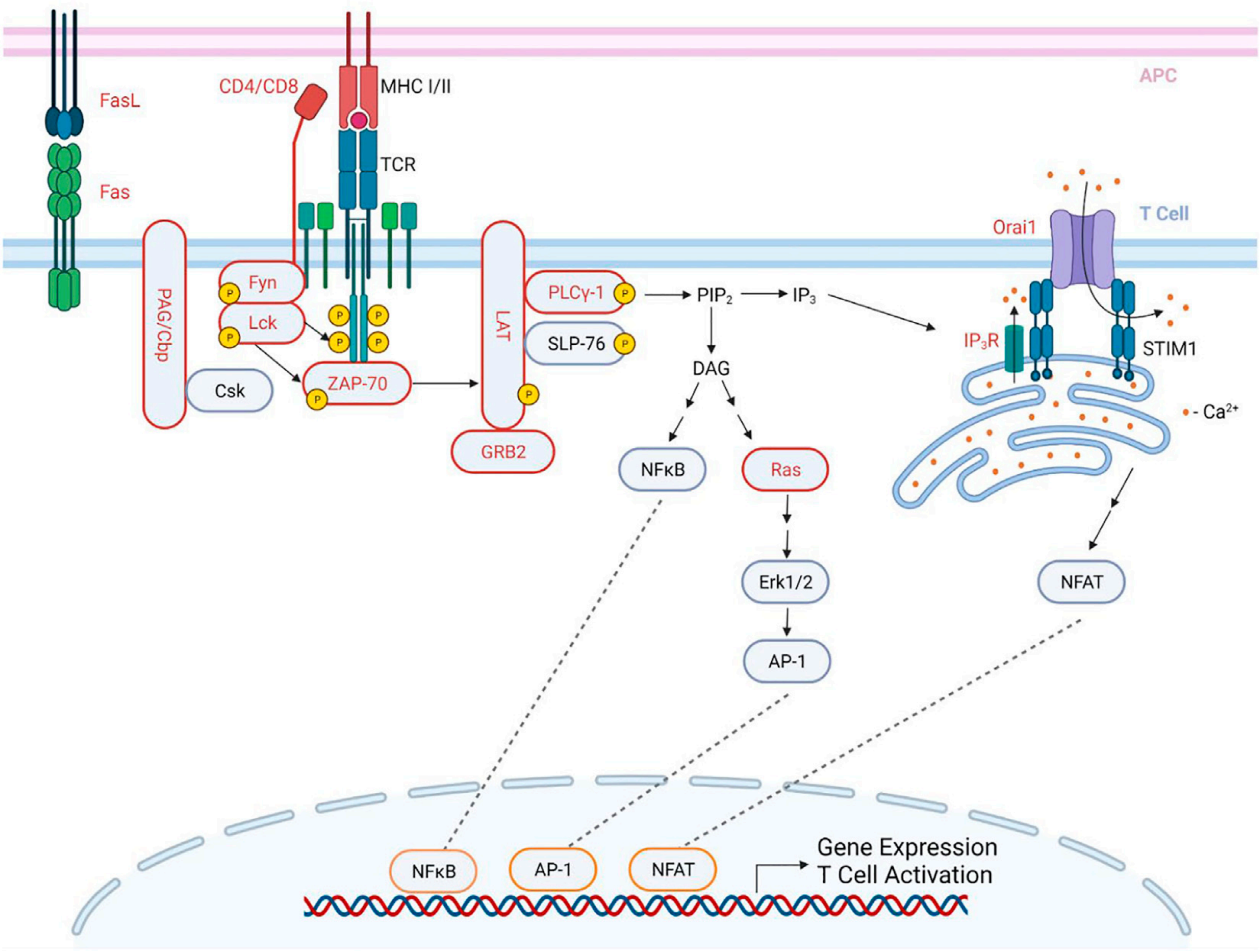
S-acylation in the proximal TCR signaling pathway. An antigen peptide, bound to an MHC of an antigen presenting cell (APC), engages and stimulates the TCR while its co-receptor (CD4/CD8) binds to the MHC, resulting in the activation of several signaling cascades, subsequently leading to T cell activation. Upon TCR stimulation, PAG/Cbp is de-phosphorylated, and disassociates from Csk, allowing for the recruitment of Lck to the TCR. Lck is then activated, and phosphorylates the ITAMs of the TCR-associated CD3 and ζ-chains. These phosphorylated ITAMs then act as a docking site for ZAP-70, which is then phosphorylated by Lck. ZAP-70 then phosphorylates and activates the scaffolding protein LAT, which is a docking site for several proteins, including SLP-76, PLC-γ1, and GRB2. These are then able to be phosphorylated and activated, many by ZAP-70. The activation of PLC-γ1 leads to the formation of IP_3_ and DAG from PIP_2_. IP_3_ then binds to the IP_3_R on the ER membrane, leading to the depletion of the ER Ca^2+^ stores. This activates STIM1, which then binds to Orai1 at ER-PM junctions, forming an activated CRAC channel, and allowing for Ca^2+^entry into the cell (shown as red dots). This then leads to the activation of the NFAT pathway. DAG activates both the NFκB pathway and the Ras pathway. These pathways result in the activation of transcription factors, ultimately leading to gene expression and T cell activation. Proteins in red are reported to be S-acylated. APC- antigen presenting cell; TCR- T cell receptor; MHC- major histocompatibility complex; ITAMs-immunoreceptor tyrosine-based activation motifs. Created with BioRender.com.

T cells are activated when the TCR and its co-receptor (CD4/CD8) recognize antigen peptides presented by the major histocompatibility complex (MHC) expressed on the antigen presenting cell (APC) (Brownlie and Zamoyska, 2013) (Figure 1). Upon TCR stimulation, Lck is activated and then phosphorylates the immunoreceptor tyrosine-based activation motifs (ITAMs) of the TCR-associated CD3 and ζ-chains (Ladygina et al., 2011; Brownlie and Zamoyska, 2013; Hwang et al., 2020). ITAMs then act as a docking site for ζ-chain associated protein-70 (ZAP-70) which is subsequently phosphorylated and activated by Lck. Activated ZAP-70 then phosphorylates LAT and SLP-76, which are critical scaffolding proteins necessary for the recruitment, both direct and indirect, of downstream effectors, such as phospholipase C-γ1 (PLC-γ1), and subsequent Ca^2+^ release from the ER stores (Smith-Garvin et al., 2009; Brownlie and Zamoyska, 2013). The proper initiation of these signaling events is required for the activation of several downstream signaling pathways, such as the NFAT, MAPK, and NF-κB pathways, which are responsible for T cell effector functions, cytokine production, cell differentiation, and cell proliferation (Ladygina et al., 2011; Brownlie and Zamoyska, 2013; Hwang et al., 2020).

These important signaling cascades are initiated at the immunological synapse (IS)—the interface between the APC and the T cell, which is highly regulated, both spatially and temporally (Bijlmakers, 2009; Dustin and Groves, 2012). Upon TCR stimulation, membrane proteins begin to reorganize into microdomains at the IS (Davis and Van Der Merwe, 2006; Ladygina et al., 2011; Dustin and Groves, 2012). These microdomains move dynamically and form concentric rings of proteins around the TCR, and the key signaling proteins are concentrated at the IS to promote T cell activation (Delon et al., 2001; Freiberg et al., 2002). A growing number of studies recognize the importance of the IS microdomains in spatial organization of pre-activated TCR signaling proteins and amplification of the specific antigenic stimuli (Burroughs et al., 2006; Seminario and Bunnell, 2008; Dustin and Depoil, 2011; Dustin and Groves, 2012; Klammt and Lillemeier, 2012). However, the mechanisms underlying antigen-induced changes in lateral movement and selective compartmentalization of the TCR signaling proteins at the IS are still not well understood.

Since lipidation is known to promote protein partitioning into the specialized liquid-ordered PM subdomains (Lucero and Robbins, 2004; Jury et al., 2007), changes in S-acylation could provide a molecular basis for protein re-organization and formation of signaling clusters at the IS upon TCR stimulation. Indeed, recent work has shown that TCR engagement leads to the S-acylation of several key T cell signaling proteins, such as ZAP-70, Lck, Orai1, and LAT, without which, these proteins do not contribute to T cell effector function (Hundt et al., 2006; Akimzhanov and Boehning, 2015; Tewari et al., 2021; Fan et al., 2020; West et al., 2022). Thus, antigen-induced protein S-acylation can be potentially explored as a novel signal transduction mechanism supporting propagation of the TCR signaling cascade at the plasma membrane.

## 3 S-ACYLATED T cell proteins

### 3.1 Receptors

#### 3.1.1 CD4/CD8

Both CD4 and CD8 are PM-localized glycoproteins that act as TCR co-receptors by binding, respectively, to class II and class I MHC ligands, and enhancing TCR activation. In 1992, Crise and Rose discovered that CD4 is S-acylated at two cysteine residues located near the junction of the transmembrane and cytoplasmic domains (Crise and Rose, 1992; Fragoso et al., 2003). They also found that the remaining three cytoplasmic cysteines are not S-acylated. However, the effect of CD4 S-acylation on its trafficking to membrane subdomains and further signaling remains controversial. Some groups state that CD4 S-acylation is required for CD4 recruitment into plasma membrane subdomains and may help to activate tyrosine phosphorylation, however, it is not sufficient to induce TCR/protein kinase C clustering and further CD4 signaling (Fragoso et al., 2003; Balamuth et al., 2004). Other groups report that S-acylation does not affect CD4 trafficking to subdomains (Del Real et al., 2002; Popik and Alce, 2004). Unfortunately, there have not been any further studies done on the effects of CD4 S-acylation on membrane subdomain recruitment and subsequent T cell effector function. More detailed studies utilizing super resolution microscopy to investigate the effects of CD4 S-acylation on its co-localization with the TCR at PM subdomains should be done. Additionally, the impact of CD4 S-acylation on T cell activation and effector function should be determined using the acylation-deficient version of the CD4 receptor.

Similar to CD4, CD8 is also S-acylated (Arcaro et al., 2000; Arcaro et al., 2001). It has been reported that the *ß* chain of CD8 is S-acylated on its cytoplasmic tail, however, a specific purpose for this S-acylation is not yet clear. One study reported that murine CD8β S-acylation supports its trafficking to PM subdomains and enhances downstream TCR signaling (Arcaro et al., 2000). However, another study found that S-acylation of human CD8β does not regulate PM subdomain targeting, but rather promotes more efficient CD8/Lck interaction and activation of Lck, and more constitutive association of CD8 with the TCR-CD3 complex (Dick et al., 2007). It has also been found that another CD8 chain, CD8α, is not S-acylated and does not partition into the PM subdomains, leading to CD8αα homodimers having reduced associations with Lck, and therefore, diminished TCR signaling, as compared to CD8αβ heterodimers (Gangadharan and Cheroutre, 2004; Dick et al., 2007). Thus, these observations indicate that S-acylation of CD8 could be used as a regulatory mechanism fine-tuning the TCR activation threshold through protein-protein interactions.

#### 3.1.2 Fas receptor

Fas receptor (FasR), also referred to as CD95, is a key transmembrane receptor for facilitating apoptosis and activation induced cell death in T cells (Zhang et al., 2001; Arakaki et al., 2014). When Fas ligand (FasL) binds to FasR, FasR oligomerizes and interacts with several other proteins, forming the death-inducing signaling complex (DISC). The DISC is then internalized, leading to the activation of effector caspases and subsequent downstream apoptotic signaling (Kaufmann et al., 2012). It has been shown that in order to induce apoptotic signaling, FasR must aggregate in PM subdomains (Kischkel et al., 1995). Chakrabandhu et al. (2007) and Feig et al. (2007) demonstrated that FasR S-acylation at Cys199 is required for its PM aggregation, and, therefore, is required for optimal apoptosis initiation. The loss of FasR S-acylation resulted in inhibited FasR internalization and suppressed activation of the downstream caspase cascade (Chakrabandhu et al., 2007; Feig et al., 2007). It has also been shown that FasL is S-acylated, as shown in a B cell lymphoma cell line (Guardiola-Serrano et al., 2010). S-acylation of FasL also enhances recruitment to PM subdomains where it can more efficiently interact with the FasR on another cell. Additionally, cells expressing acylation-deficient FasL had significantly lower levels of soluble FasL (Guardiola-Serrano et al., 2010). Overall, S-acylation of both FasR and its ligand leads to enhanced Fas-mediated apoptosis, while a loss of S-acylation in either protein impairs apoptosis (Guardiola-Serrano et al., 2010). FasR and FasL are important examples demonstrating a critical role of S-acylation in regulation of the receptor function, and its significant physiological outcomes.

### 3.2 Kinases

#### 3.2.1 Lck

Lymphocyte-specific protein tyrosine kinase (Lck) is a member of the Src family of non-receptor tyrosine kinases that plays a critical role in early stages of T cell activation. Upon antigen stimulation, Lck is localized to the TCR-CD3 complex where it phosphorylates the ITAMs and, subsequently, activates recruited ZAP-70 to promote the signaling cascade (Lo et al., 2018). The kinase activity of Lck is largely controlled by phosphorylation and dephosphorylation of the tyrosine residue within the catalytic domain, and a significant fraction of Lck is constitutively activated even in quiescent T cells (Boggon and Eck, 2004; Nika et al., 2010). Interestingly, the amount of activated Lck did not change upon TCR engagement (Nika et al., 2010), indicating that dynamic regulation of Lck activity during TCR signaling is not achieved solely through its phosphorylation, but rather supported by a different mechanism.

In 1993, it was found that Lck is N-myristoylated at Gly2 and dually S-acylated at Cys3 and Cys5 (Paige et al., 1993). Although the individual impact of these cysteines is debated, it has been suggested that S-acylation of Lck, but not N-myristoylation, is required for its recruitment to the plasma membrane and propagation of TCR signaling (Yurchak and Sefton, 1995; Kabouridis et al., 1997). Using subcellular fractionation and confocal microscopy, Kabouridis, et al. (1997) demonstrated that without dual S-acylation Lck does not fully localize to the PM. Another group used total internal reflection fluorescence microscopy (TIRFM) to find that acylation-deficient Lck mutants were not detectable at the PM of Jurkat cells (Zimmermann et al., 2010). However, we have shown, using confocal microscopy, that S-acylation-deficient Lck does still localize to the Jurkat cell PM (Akimzhanov and Boehning, 2015). Despite these contrasting localization results, all of these groups concluded that Lck S-acylation is required for its proper function. Despite acylation-deficient mutants of Lck being still catalytically active (indicated by an *in vitro* assay), they failed to promote a calcium response upon TCR stimulation resulting in loss of NFAT activation, and lack of IL-2 cytokine production and CD69 surface expression, important markers of T cell activation (Kabouridis et al., 1997). Importantly, S-acylation of Lck is agonist-dependent; stimulation of either T cell or Fas receptors triggers extremely rapid (within minutes) and transient increase in S-acylation of Lck (Akimzhanov and Boehning, 2015; Fan et al., 2020; Bieerkehazhi et al., 2022). These findings suggest that activity of Lck in stimulated T cells could be regulated by S-acylation which serves as a molecular basis for dynamic spatial reorganization of pre-activated Lck molecules. Furthermore, this model implies that two classes of enzymes, zDHHC PATs and protein thioesterases, could be recognized as an essential part of the proximal TCR signaling machinery.

#### 3.2.2 Fyn

Fyn, another member of the Src kinase family, is also involved in the early stages of T cell activation and has many of the same phosphorylation targets as Lck. Similar to Lck, Fyn is both N-myristoylated and dually S-acylated. It was found that, like Lck, N-myristoylation of the N-terminal glycine residue is a prerequisite for S-acylation at Cys3 and Cys6 (Alland et al., 1994). It is likely that N-myristoylation of Fyn increases accessibility to a Fyn-specific protein acyltransferase, allowing for the addition of two acyl chains to the protein. Although Fyn can be S-acylated at two cysteine residues, it has been shown that the majority of Fyn protein is singly S-acylated at Cys3, and lipidation of this residue is sufficient for protein localization to the PM and further partitioning into the specialized PM subdomains, such as the IS (Van ’t Hof and Resh, 1997; Honda et al., 2000; Liang et al., 2001). Similar to Lck, the lack of PM-associated Fyn also results in decreased calcium fluxes and cell proliferation upon TCR activation, but is much less severe than that of S-acylation deficient Lck, probably due to a significant degree of redundancy between the two kinases (Ladygina et al., 2011). An important distinction of Fyn and Lck regulation is the requirement of N-myristoylation prior to S-acylation, illustrating a possible interplay between S-acylation and other types of protein lipidation.

#### 3.2.3 ZAP-70

ZAP-70 is a key signaling protein of the proximal TCR pathway. Upon TCR activation, it is recruited from the cytoplasm to ITAMs where it is phosphorylated by Lck. Activated ZAP-70 is then released from ITAMs and proceeds to target its effectors, primarily membrane-bound scaffolding proteins LAT and SLP-76 (Courtney et al., 2018). Recently, our group demonstrated TCR-dependent S-acylation of ZAP-70 at a single cysteine residue, Cys564, localized within the C-terminal part of the protein (Tewari et al., 2021). Interestingly, a homozygous mutation of this lipidation site (c.1690T>C; p. C564R) was reported to be associated with severe combined immunodeficiency in a human patient (Ren et al., 2008), indicating that impaired S-acylation of T cell proteins could be implicated in a pathogenesis of immune system disorders.

Similar to Lck, the acylation-deficient mutant of ZAP-70 was still catalytically active, however, it failed to phosphorylate its substrates resulting in disrupted TCR signaling thus preventing T cell activation (Tewari et al., 2021). Interestingly, biochemical and imaging approaches showed that loss of S-acylation did not disrupt ZAP-70 recruitment to the PM and subsequent activation by Lck (Tewari et al., 2020b). Although the reason why the acylation-deficient ZAP-70 was still unable to phosphorylate its downstream targets is not entirely clear, these observations indicate that TCR-induced S-acylation can mediate translocation of the activated ZAP-70 into spatially segregated PM subdomains containing its downstream effectors. In this scenario, PM recruitment and activation of ZAP-70 is a prerequisite for a zDHHC-mediated S-acylation that drives its subsequent migration into the LAT and SLP-76-containing lateral compartments.

### 3.3 Adaptor proteins

#### 3.3.1 LAT

After phosphorylation by ZAP-70, linker for activation of T cells, LAT, acts as a docking site for several T cell signaling proteins, such as SLP-76 and phospholipase Cγ-1 (PLC-γ1). The binding of these proteins to LAT is required for their proper activation and subsequent signaling. LAT is a transmembrane protein and requires S-acylation at Cys26 and Cys29 for its localization to the PM and its interaction with the TCR (Zhang et al., 1998; Tanimura et al., 2006). Upon isolation of the IS, it was found that the TCR and S-acylated LAT form a scaffold for TCR signal transduction proteins, while acylation-deficient LAT was unable to co-localize with the TCR (Harder and Kuhn, 2000), due to its retention to the Golgi (Tanimura et al., 2006). Additionally, S-acylation of LAT and subsequent partitioning into PM subdomains is essential for efficient LAT activation, as S-acylation deficient LAT mutants were substantially less phosphorylated compared to WT LAT upon TCR stimulation in Jurkat cells (Zhang et al., 1998; Tanimura et al., 2006). S-acylation was also shown to support stability of LAT protein, as acylation-deficient LAT is more susceptible to premature degradation than WT LAT, suggesting that LAT S-acylation contributes to protein stability (Tanimura et al., 2006). Importantly, it has also been shown that impairment of LAT S-acylation in murine CD4^+^ T cells was linked to a state of functional unresponsiveness known as T cell anergy, further supporting that S-acylation of T cell proteins has important physiological implications (Hundt et al., 2006).

#### 3.3.2 PAG/cbp

Phosphoprotein associated with GEMs (glycosphingolipid-enriched microdomains) (PAG)/Csk-binding protein (Cbp) is another S-acylated T cell adaptor protein (Posevitz-Fejfár et al., 2008). In resting T cells, Fyn-phosphorylated PAG/Cbp recruits Csk to the PM (Yasuda et al., 2002). When bound to PAG/Cbp, Csk inhibits Src family kinases, Lck and Fyn through phosphorylation of carboxyl terminal tyrosine residues. Upon TCR stimulation, PAG/Cbp becomes dephosphorylated and dissociates from Csk. Lck and Fyn are then dephosphorylated by the protein tyrosine phosphatase CD45 and are able to be activated through phosphorylation of tyrosine residues within their respective kinase domains (Brdicka et al., 2000; Davidson et al., 2016). As a negative regulator of Lck and Fyn, PAG/Cbp plays an important role in preventing aberrant T cell activation (Hořejší, 2004).

Using radiolabeled palmitate, it was found that PAG/Cbp is S-acylated (Brdicka et al., 2000). This protein has a CxxC motif next to its transmembrane domain, making those two cysteines the most likely sites for S-acylation, however, the S-acylation site has not yet been confirmed using acylation assays. Mutating the CxxC to AxxA resulted in a presumably acylation-deficient PAG/Cbp which was used to investigate the effects of lipidation on PAG/Cbp function (Posevitz-Fejfár et al., 2008). Under normal circumstances, PAG/Cbp localizes to GEMs. While PAG/Cbp (AxxA) maintains localization to the PM, it does not localize to the GEMs. However, it is still phosphorylated by Fyn and recruits Csk to non-GEM fractions, further confirming that PAG/Cbp-Csk binding is a phosphorylation-dependent event (Kischkel et al., 1995). Although PAG/Cbp (AxxA) still binds to Csk, this mutation does affect the protein function. It was found that the AxxA mutant results in increased Lck and ZAP-70 phosphorylation, however it is not yet known if this is due to PAG/Cbp being constitutively active when localized to non-GEM domains. Lastly, Jurkat T cells over-expressing PAG/Cbp (AxxA) experienced enhanced migration in response to stromal cell-derived factor 1 (SDF-1), which is reported to preferentially signal from within GEMs (Nguyen and Taub, 2002; Posevitz-Fejfár et al., 2008). These data highlight the importance of PAG/Cbp and Csk recruitment to GEMs, and suggest that S-acylation plays an important role in this protein recruitment and further downstream signaling.

### 3.4 Ion channels

#### 3.4.1 IP3R

The inositol 1,4,5-trisphosphate receptor (IP_3_R) is an ER-localized Ca^2+^ channel playing a critical role in the initiation of the TCR pathway. Engagement of the TCR leads to PLC-γ1 activation and production of IP_3_ which binds and activates the IP_3_R channel. IP_3_R-mediated depletion of the ER Ca^2+^ stores triggers store-operated Ca^2+^ entry (SOCE) and, consequently, orchestrates a variety of downstream signaling events in T cells (Hirota et al., 1998; Trebak and Kinet, 2019). Mass spectrophotometry of the IP_3_R protein revealed S-acylation of two cysteines (Cys56, 849) and bioinformatic analysis predicted S-acylation of a third residue (Cys2214). Decreased S-acylation of the IP_3_R resulted in reduced Ca^2+^ influx, likely due to premature lysosomal degradation and decreased protein expression, but did not affect localization to the ER (Fredericks et al., 2014). This same study found that IP_3_R is S-acylated by zDHHC6 which forms a complex with selenoprotein K (SelK), further discussed below. Interestingly, prior to this discovery, this same group found that SelK KO mice have reduced T cell calcium fluxes in response to αCD3 and chemokine stimulation, as well as reduced T cell proliferation and migration, presumably due to the lack of IP_3_R S-acylation (Verma et al., 2011). These data taken together suggest that S-acylation regulates the function of IP_3_R through stabilizing protein expression.

#### 3.4.2 Orai1/STIM1

SOCE is a central component of intracellular signaling pathways, including TCR responses in T cells. It is triggered upon receptor engagement and consequent depletion of ER Ca^2+^ stores thorough the IP_3_R Ca^2+^ channel. In T cells, SOCE is mediated by the Ca^2+^ release-activated calcium (CRAC) channels formed primarily by STIM1 and Orai1 proteins (Feske et al., 2006; Soboloff et al., 2006; Vig et al., 2006; Zhang et al., 2006). STIM1 serves as a Ca^2+^ sensor that detects depletion of the ER stores *via* EF hands on the luminal portion of the protein. Upon store depletion, STIM1 undergoes a conformational change which allows its SOAR/CAD activation domain to bind Orai1 and gate the channel to allow Ca^2+^ entry (Kim and Muallem, 2011). Both Orai1 and STIM1 are critical for T cell immunity as evident from loss of function mutations of Orai1 or STIM1 which eliminate SOCE and result in impaired cytokine production, abrogated T cell activation, and severe combined immunodeficiency in patients (Feske et al., 2006; Gwack et al., 2008; Oh-Hora et al., 2008; Picard et al., 2009).

The critical step in activation of the CRAC channel is STIM1 translocation into large clusters (“puncta”) at ER-plasma membrane junctions where it interacts with Orai1 subunits (Liou et al., 2005; Stathopulos et al., 2006; Pani et al., 2008; Stathopulos et al., 2008; Shaw et al., 2013; Yazbeck et al., 2017). In particular, the enhanced localized Ca^2+^ influx was observed at the T cell/APC contact area, indicating that in T cells Orai1/STIM1-mediated SOCE preferentially occurs within the IS (Barr et al., 2008; Lioudyno et al., 2008; Cahalan and Chandy, 2009). Using acyl-biotin exchange (ABE)-based screening, we found that both major components of the T cell CRAC channel, Orai1 and STIM1, are S-acylated proteins (West et al., 2022). We found that TCR stimulation results in rapid increase in Orai1 S-acylation at Cys143, and that modification of this residue is critical for Orai1 co-localization with STIM1, puncta formation, and SOCE (West et al., 2022). Our findings were independently confirmed by another group which showed that lack of Orai1 S-acylation also decreased NFATC1 translocation to the nucleus and led to diminished IL-2 production in Jurkat T cells (Carreras-Sureda et al., 2021). Similarly, we found that STIM1 undergoes TCR-dependent S-acylation (Kodakandla et al., 2022). Disruption of the STIM1 S-acylation site (Cys437) did not affect its ER localization, but was critical for the assembly of STIM1 into puncta with Orai1 and proper CRAC channel formation. Altogether, these data demonstrate that dynamic stimulus-dependent S-acylation of CRAC channel components is a critical regulator of Ca^2+^ signaling in T cells, and, consequently, can have significant implications for T cell function.

### 3.5 Other proteins

#### 3.5.1 Ras

Ras proteins are ubiquitously expressed small GTPases critically regulating a variety of T cell processes including T cell development, differentiation, and function (Ladygina et al., 2011; Lapinski and King, 2012). Upon TCR stimulation, Ras proteins are activated by GTP exchange and promote downstream signaling cascades leading to T cell activation and proliferation (Downward et al., 1990; Ebinu et al., 2000; Genot and Cantrell, 2000; Lapinski and King, 2012). Although three Ras isoforms, H-Ras, K-Ras and N-Ras, are closely related and share conserved effector binding domains, their hypervariable C-terminal regions are subjected to different lipid modifications. All isoforms are irreversibly modified with a 15-carbon farnesyl moiety, however, only H-Ras, N-Ras and a K-Ras4B splice variant of K-Ras are S-acylated (Eisenberg et al., 2013). While farnesylation itself is not sufficient for a stable membrane association, additional single (N-Ras and K-Ras4B) or dual (H-Ras) S-acylation promotes Ras recruitment from endomembranes such as the Golgi and ER through vesicular transport, and PM association of non-acylated K-Ras4B is supported by an electrostatic interaction of its polybasic motif (Hancock et al., 1990; Rubio et al., 2010). The ability of Ras to undergo dynamic cycles of S-acylation and de-acylation plays the essential role in regulation of Ras signaling function. S-acylation increases Ras recruitment the PM, and de-acylation promotes rapid retrograde trafficking back to the Golgi, thus preventing Ras over-activation (Rocks et al., 2003). Acylation-deficient N/H-Ras mutants are retained in endomembranes and are not activated upon TCR stimulation (Rubio et al., 2010). Interestingly, non-acylated K-Ras4B is targeted to into cholesterol-independent PM subdomains that do not overlap with H-Ras partitions (Niv et al., 2002; Prior et al., 2003), further supporting the observation that S-acylation can serve as a molecular basis for selective signaling at the PM.

#### 3.5.2 STAT3

Signal transducer and activator of transcription 3 (STAT3) is another prime example of a protein who’s function is regulated by the S-acylation/de-acylation cycle. This transcription factor is recruited to the PM upon activation of cytokine receptors where it is phosphorylated by JAK2, a member of the Janus activating kinases family. Activated STAT3 is then translocated to the nucleus where it induces transcription of its target genes, including *RORC* and *IL17A* which drive differentiation of the T helper 17 (Th17) cells (Capone and Volpe, 2020). Recently, Zhang, et al. (2020) found that STAT3 is S-acylated at Cys108. They determined that the S-acylation of STAT3 is required for the PM recruitment and activation by JAK2, however, phosphorylated STAT3 needs to be de-acylated to be translocated to the nucleus. Even though STAT3 was still able to form homodimers and was present in the nucleus, disruption of the S-acylation/de-acylation cycle of STAT3 in mouse splenocytes prevented its PM recruitment, resulting in inhibited Th17 differentiation (Zhang et al., 2020). This study reveals a unique mechanism controlling STAT3 signal transduction and indicates that S-acylation can potentially serve as a molecular switch between the classical transcriptional function of STAT3 and its non-canonical role in the cytoplasm. Since aberrant function of STAT3 is associated with a variety of human disorders, such as inflammatory diseases and cancer, this study further highlights the therapeutic potential of targeting S-acylation.

#### 3.5.3 VAMP7

VAMP7 belongs to a ubiquitously expressed family of SNARE proteins known as mediators of the vesicular fusion. In T cells, SNAREs have been shown to be involved in TCR recycling to the IS, and it has been found that VAMP7 is particularly important for T cell activation as it is required for LAT recruitment to TCR activation sites, LAT phosphorylation, and downstream T cell signaling (Larghi et al., 2013). It has been shown that S-acylation of VAMP7 at Cys183 is required for its localization to the Golgi under resting conditions. Acylation-deficient VAMP7 instead localizes to the cytosol, presumably in vesicles, and is not properly recruited to the IS upon TCR activation (Morrison et al., 2020). Although the effects of VAMP7 mislocalization on activation of LAT were not assessed in the study, it is likely that loss of VAMP7 S-acylation has significant consequences for downstream TCR signaling.

## 4 zDHHC enzymes in T cells

The zDHHC family of protein acyltransferases are typically four-pass transmembrane proteins with the zDHHC (Asp-His-His-Cys) catalytic sequence and the C-terminal tail located within the cytosol. The majority of zDHHC acyltransferases were shown to be localized to the Golgi and endoplasmic reticulum (ER) membranes; others were identified as PM- and ER-localized enzymes (Gorleku et al., 2011; Abrami et al., 2017). However, localization of zDHHC enzymes was in many cases examined using proteins overexpressed in HEK293 and HeLa cell lines, therefore, further studies should be done to verify localization of endogenously expressed proteins (Ohno et al., 2006).

**FIGURE 2:**
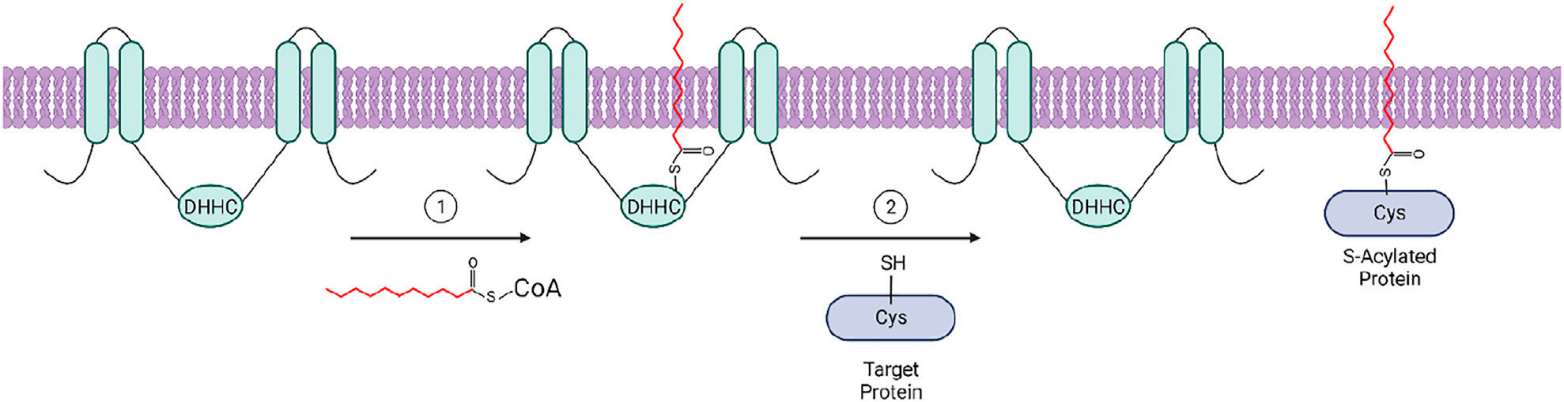
The two-step “ping pong” mechanism of zDHHC-mediated protein S-acylation: 1) The acyl group from an acyl-CoA molecule is transferred to the catalytic cysteine residue of the ZDHHC enzyme within the cytosolic loop between transmembrane domains two and three, forming an acyl-enzyme intermediate. 2) The acyl group is subsequently transferred to a free cysteine residue of a target protein, resulting in an S-acylated protein. Created with BioRender.com.

A 2012 study revealed the “ping-pong” enzymatic mechanism of the S-acylation reaction indicating that zDHHC enzymes employ a two-step mechanism to modify their protein substrates (Jennings and Linder, 2012). During the first step, an acyl group from acyl-CoA is first transferred to the cysteine residue within the catalytic core of the zDHHC enzyme, forming an acyl-enzyme intermediate. This is then followed by the transfer of the acyl group to a cysteine on the target protein (Figure 2) (Jennings and Linder, 2012). Consistent with the diversity of acyl chains added to substrate proteins, zDHHCs also show some level of acyl chain specificity, likely determined at the acyl-enzyme intermediate step. The first evidence of lipid substrate specificity among these enzymes was reported by Jennings and Linder (Jennings and Linder, 2012), who observed a difference in acyl-CoA preferences between zDHHC3 and zDHHC2 in a competition assay. They found that zDHHC2 is capable of transferring palmitate (C16:0) and longer fatty acids (C18:0, C20:0, C20:4) to a substrate peptide with comparable efficiency, while zDHHC3 showed strong preference for C14 and C16 fatty acids. A more recent study used chemically synthesized azide and alkyne fatty acid probes to compare the lipid substrate specificity of zDHHC3 and zDHHC7 (Greaves et al., 2017). Although these enzymes share close similarity in the primary sequence, the study showed that zDHHC7 has a greater ability to incorporate C18:0 chains compared with zDHHC3. Importantly, the preference for shorter fatty acids was linked to a single amino acid, Ile182, localized in the transmembrane domain 3 of zDHHC3 (Greaves et al., 2017). This indicates that the selectivity of zDHHCs is not decided by availability of the Acyl-CoA substrate, but likely defined at the structural level. This observation is supported by the recent insights into the structure and mechanisms of two closely related enzymes, zDHHC15 and zDHHC20, which revealed that the fatty acyl chain fits into a cavity composed of the four transmembrane helices (Rana et al., 2018a). The residues lining this cavity define its chemical properties and, consequently, the ability to accommodate fatty acyl chains of different length. The sequence analysis of the zDHHC members indicates that the cavity lining residues vary between different zDHHC enzymes indicating that the fatty acyl chain selectivity of zDHHC enzymes is likely determined at the structural level, rather than availability of Acyl-CoA species (Rana et al., 2018b). Although the biological significance of differential fatty S-acylation is still not fully understood, it is possible that attachment of different fatty acid species can selectively drive proteins into PM domains with various degrees of membrane order. It has been shown that when cells are incubated with polyunsaturated fatty acids (PUFAs), Fyn is S-acylated with those PUFAs, resulting in decreased localization to PM subdomains and decreased MAP kinase activation (Liang et al., 2001). It is not known if this happens in a more physiologically relevant context, but does illustrate the ability of zDHHC enzymes to S-acylate proteins with fatty acids other than the often-studied palmitic acid. Thus, zDHHC protein acyltransferases could serve as enzymes controlling the threshold of T cell activation by targeting signaling proteins in and out of TCR microdomains.

The following section describes PATs that have known roles in T cell signaling. While this is not a full list of zDHHCs expressed in T cells, it does describe zDHHC enzymes known to S-acylate key T cell proteins.

### 4.1 DHHC6

zDHHC6, permanently localized to the ER membrane, is implicated in T cell signaling due to its ability to S-acylate and stabilize the IP_3_R (Fredericks et al., 2014). In Jurkat T cells, shRNA-induced knockdown of zDHHC6 reduced S-acylation and protein expression of the IP_3_R leading to decreased Ca^2+^ flux upon TCR stimulation (Fredericks et al., 2014). It was also found that in order to facilitate S-acylation of the IP_3_R, zDHHC6 forms a complex with an ER-localized selenium-containing selenoprotein K (SelK), a protein previously reported to be involved in regulation of SOCE (Fredericks and Hoffmann, 2015; Fredericks et al., 2018). Similarly, SelK promotes S-acylation of CD36, the fatty acid transporter, also known to play important roles in T cell biology (Zamora et al., 2013; Wang et al., 2020; Xu et al., 2021). S-acylation of CD36 was shown to be mediated by zDHHC5, however, it is unclear whether SelK forms a complex with zDHHC5 as well. In addition to SelK, the enzymatic function of zDHHC6 can be regulated through the S-acylation of its SH3 domain by another protein acyltransferase, zDHHC16, suggesting the existence of S-acylation cascades (Fredericks et al., 2014; Abrami et al., 2017). Interestingly, S-acylation of zDHHC6 can occur at three different sites leading to potentially eight different zDHHC6 species defined by acyl site occupancy (Abrami et al., 2017), indicating that localization, stability and function of zDHHC6 can be fine-tuned through the actions of upstream protein acyltransferases and thioesterases.

### 4.2 zDHHC7

zDHHC7 mediates S-acylation of two proteins playing prominent roles in regulation of the T cell function: the FasR and STAT3. zDHHC7 knockdown reduced both total expression of the FasR and its PM localization preventing proper activation of Fas-induced cell death (Rossin et al., 2015). At the molecular level, zDHHC7-mediated S-acylation supports optimal cell surface expression of FasR by protecting it from premature lysosomal degradation, thus maintaining cellular sensitivity to apoptotic signals (Chakrabandhu et al., 2007; Rossin et al., 2015).

zDHHC7 is also responsible for the S-acylation of STAT3 as evidenced by a lack of STAT3 S-acylation in zDHHC7 knockout cells (Zhang et al., 2020). Furthermore, re-expression of zDHHC7 in the knockout cells resulted in a significant increase in STAT3 S-acylation that was not present when the knockout cells expressed a catalytically inactive form of zDHHC7. Since increased STAT3 activation and Th17 cell population are markers of inflammatory bowel disease (IBD) (Britton et al., 2019), the expression of *Zdhhc7* and STAT3 target genes were studied in patients with IBD. It was found that, compared to healthy individuals, patients with IBD have increased *Zdhhc7* mRNA, pointing to a correlation between dysregulated STAT3 S-acylation and IBD (Zhang et al., 2020). To further investigate this, the authors used *Zdhhc7* knockout mice in an intestinal colitis model, and found that these mice had reduced Th17 cell differentiation and were somewhat protected from colitis. This study highlights the importance of zDHHC enzymes as potential therapeutic targets for IBD and other inflammatory diseases (Zhang et al., 2020).

### 4.3 zDHHC21

Our group has found that PM-localized zDHHC21 is a critical signaling protein in both human and murine T cells. In Jurkat T cells, zDHHC21 knockdown resulted in the inhibition of Fas-mediated phosphorylation of Lck, ZAP-70, and LAT, as well as the inhibition of calcium release through the IP_3_R after FasL stimulation (Akimzhanov and Boehning, 2015). Stimulation of EL-4 murine T cells with cross-linked αCD3/CD28 led to an increased association of zDHHC21 with both Lck and Fyn, while zDHHC21 knockdown led to decreased Lck and Fyn S-acylation, suggesting that zDHHC21 interacts with and either directly or indirectly S-acylates Lck and Fyn (Fan et al., 2020). However, it should be noted that another group suggests that zDHHC2 is responsible for Lck S-acylation (Zeidman et al., 2011). Given that zDHHC enzymes often demonstrate a significant degree of promiscuity (Huang et al., 2009; Greaves and Chamberlain, 2011), it is possible that both zDHHC2 and zDHHC21 could play a role in Lck S-acylation. Furthermore, zDHHC21 knockdown in primary naive murine CD4^+^ T cells resulted in decreased effector cytokine production, as well as cellular activation and differentiation (Fan et al., 2020). These results indicate a requirement of zDHHC21 for proper activation of TCR signaling proteins and T cell effector function.

The depilated mouse strain (*Zdhhc21* ^*(dep/dep)*^; dep mice) further demonstrates the importance of zDHHC21 (Mayer et al., 1093). Dep mice have a spontaneous recessive three base pair deletion in the *Zdhhc21* gene resulting in an in-frame deletion of Phe233 of zDHHC21. This mutation results in decreased enzymatic activity without significantly affecting expression levels of the enzyme (Mayer et al., 1093; Mill et al., 2009). The depilated mutation results in a loss of Fyn S-acylation, and therefore, mislocalization. Since Fyn is a key signaling protein in keratinocyte development, this affects the *epidermis* and causes disoriented, fragmented, and misshapen hair follicles and shafts, resulting in the gross phenotype of thin, short, matted, and greasy hair, giving the mouse strain the name, depilated (Mayer et al., 1093). Since zDHHC21 is expressed in several different cell types, the dep phenotype goes beyond just the *epidermis*. Dep mice also experience endothelial dysfunction and decreased endothelial inflammation after injury, indicating the functional effects of this zDHHC21 mutation (Beard et al., 2016).

Recently, we found that the dep phenotype extends to T cells as well (Bieerkehazhi et al., 2022). The deletion of F233 in zDHHC21 causes the loss of a canonical Ca^2+^/calmodulin binding motif, which disrupts calmodulin binding to the C-terminal tail of zDHHC21 in dep CD4^+^ T cells. This suggests that the dysfunctional nature of ΔF233 zDHHC21 is likely due to the inability of the mutated enzyme to respond to stimulation-induced Ca^2+^ release, suggesting that it is regulated by changes in cytosolic Ca^2+^ concentration and subsequent calmodulin binding. When testing to determine if ΔF233 zDHHC21 affects S-acylation of zDHHC21 substrates, we found that under resting conditions there were no differences in S-acylation of signaling proteins between CD4^+^ T cells from WT and dep mice. However, when the cells were co-stimulated with αCD3/CD28, WT cells showed an increase in Lck, PLC-γ1, and Erk1/2 S-acylation, while dep CD4^+^ T cells showed no changes in S-acylation. This indicates that zDHHC21 is required for TCR stimulation-induced S-acylation, but not basal S-acylation. Additionally, it was found that dep CD4^+^ T cells had reduced phosphorylation of Lck, ZAP-70, LAT, Erk1/2, PLC-γ1, Jnk, and p38 upon TCR stimulation. Consistent with decreased PLC-γ1 phosphorylation, dep T cells also had reduced calcium release from the ER stores upon stimulation with anti-CD3. Lastly, we found that ΔF233 zDHHC21 does not affect T cell development, but it does disrupt T cell activation and differentiation into Th subtypes, as evidenced by decreased cytokine production and expression of transcription factors specific to Th1, Th2, and Th17 lineages (Bieerkehazhi et al., 2022). These data together, suggest that zDHHC21-mediated S-acylation may be one of the earliest signaling events after TCR stimulation, and demonstrate that zDHHC21-mediated S-acylation is a key part of the early TCR signal transduction cascade. Due to the changes in stimulation-induced S-acylation but not basal S-acylation observed in dep mice, ΔF233 zDHHC21 provides an opportunity to test the effects of transient agonist-induced S-acylation on cell signaling, which is not possible in conventional knockdown and knockout models.

## 5 Conclusion

Despite the early discovery of protein S-acylation in 1979 (Schmidt and Schlesinger, 1979), its role in the immune system remained largely unexplored. Recent development of robust biochemical techniques to detect protein S-acylation, frequently coupled with advanced proteomics approaches, resulted in a rapidly growing number of newly discovered S-acylated proteins. Among them, there is a broad variety of proteins with pivotal roles in T cell signaling, including receptors (CD4, CD8, FasR), enzymes (Lck, ZAP-70, PLC-γ1), adaptor proteins (LAT, PAG/Cbp), calcium channels (Orai1, IP_3_R), and even a transcription factor (STAT3). Given the significant impact of S-acylation on protein function, these findings indicate that S-acylation is a widespread post-translational modification playing a vital role in the T cell-mediated immune responses.

The addition of a long-chain fatty acid moiety is known to affect either protein localization to the PM, or, while at the PM, partitioning into liquid ordered, cholesterol and sphingolipid enriched PM fractions, functionally designated as PM domains or lipid rafts. These domains, including the immunological synapse in T cells, serve to confine signaling proteins and, consequently, promote specific protein-protein interactions. Although kinetics of S-acylation is not often assessed, several examples, such as kinases Lck and ZAP-70, or CRAC channel proteins, demonstrate the extremely dynamic nature of S-acylation in T cells. These observations indicate a potentially important function of S-acylation as a signal transduction mechanism mediating rapid assembly of the TCR signaling machinery at the IS in response to antigenic stimulation.

S-acylation of Lck was discovered more than two decades ago. However, until recently, enzymes that catalyze S-acylation of Lck and other T cell proteins, remained enigmatic. Although we are just beginning to understand the role of zDHHC enzymes in T cells, recent reports showing that zDHHC21 and zDHHC7 are critical for Th differentiation demonstrate that characterization of protein acyltransferases can potentiate the discovery of novel immunoregulatory pathways. These early findings provide first evidence that zDHHC enzymes can play critical functions in regulation of the adaptive immune system.

Much more work still needs to be done to further identify and characterize S-acylated T cell proteins, and define the role of zDHHC protein acyltransferases and thioesterases in regulation of T cell function. However, we hope that this review provides a sufficient insight into the importance of protein S-acylation for T cell function and demonstrates that this modification and mediating enzymes could be promising therapeutic targets in the diseases associated with altered T cell homeostasis.
